# The Effects of Mindfulness-Based Cognitive Therapy on Depression and Anxiety in Women with Premenstrual Syndrome

**DOI:** 10.1155/2016/9816481

**Published:** 2016-11-29

**Authors:** Faeze Panahi, Mahbobeh Faramarzi

**Affiliations:** ^1^Department of Psychology, Islamic Azad University, Ayatollah Amoli Branch, Amol, Iran; ^2^Infertility and Reproductive Health Research Center, Health Research Institute, Babol University of Medical Sciences, Babol, Iran

## Abstract

*Objective*. Little research has been done regarding the role of psychotherapy in the treatment of Premenstrual Syndrome (PMS). The aim of this study was to examine the effect of mindfulness-based cognitive therapy (MBCT) on the PMS symptoms and depression and anxiety symptoms in women with PMS.* Design*. In a randomized controlled trial, a total of 60 students at Mazandaran University with mild to moderate PMS who had depressive symptoms (Beck depression scores 16–47) were randomly allocated to either an experimental (*n* = 30) or a control (*n* = 30) group. The experimental group received MBCT in eight group sessions (120 min each) over 8 weeks. The control group received no intervention. All participants completed the Premenstrual Assessment Scale (PAS), Beck Depression Inventory (BDI), and Beck Anxiety Inventory (BAI) at the beginning and the end of the study. Repeated-measure ANOVA was used to analyze the data.* Results*. At the end of study, the experimental and control groups showed the following scores, respectively (mean ± SD): depression, 15.73 ± 6.99 and 25.36 ± 7.14; anxiety, 16.96 ± 7.78 and 26.60 ± 9.38; and total PAS, 42.86 ± 8.02 and 58.93 ± 8.47. MBCT improved depression and anxiety symptoms and total PAS score.* Conclusion*. MBCT intervention is acceptable and potentially beneficial in women with PMS symptoms. Psychotherapy should be considered as a treatment option for mild to moderate PMS in women with depressive symptoms.

## 1. Introduction

Premenstrual Syndrome (PMS) has various psychological (anxiety, depression, hostility, poor concentration, confusion, social disturbance, and interpersonal conflicts) and physical (insomnia or hypersomnia, headache, pelvic pain and discomfort, breast tenderness, joint pain, and feeling overwhelmed) symptoms that recur regularly beginning 7–14 days before the onset of menstruation [1]. Approximately 75% of reproductive age women experience some PMS symptoms [2]. A small proportion of women (3%–8%) have an extreme form of psychological manifestations known as premenstrual dysphoric disorder (PMDD) [3]. PMS can interfere with normal activity, as well as interpersonal relationships [4]. Women with PMS reported a poorer quality of life and required increased pharmacological treatment [5]. Farrokh-Eslamlou et al. showed that quality of life, particularly psychological and social components, mental health, and environmental health, decreased in medical students with PMS [6]. Premenstrual distress also tends to develop in the context of women's life, especially in the context of intimate relationships. There is convincing evidence that women with severe PMS have fewer relationships [7, 8]. Thus, PMS has a negative impact on the relationship of women with their partners and children, as well as in domestic responsibilities [7, 9].

Research has demonstrated the influence of psychological processes on PMS. Psychological factors such as alexithymia, neurotic personality, and high anxiety are predictors of PMS and pelvic pain [10, 11]. It has long been established that PMS is frequently comorbid with psychiatric disorders [12]. A previous study highlighted the association between the severity of PMS and psychiatric symptoms [13]. Unfortunately, the treatment of PMS remains a major challenge. The symptomatic improvement of patients with mild to moderate PMS and pelvic pain after pharmacological intervention remains controversial [14, 15]. Pharmacologic therapies carry a greater risk of adverse events and should only be offered to patients with persistent symptoms [16]. According to the American College of Obstetricians and Gynecologists, nonpharmacotherapy is indicated as a first-line treatment option for less severe PMS [17].

Although some research has shown that pharmacotherapies are not more effective as treatment than cognitive behavioral therapy (CBT) in patients with depression [18–20], little research is available regarding the role of CBT in PMS [21, 22]. Lustyk et al. showed that CBT was effective in the management of PMS [23], and Busse et al., in a meta-analysis of nine randomized trials, concluded that CBT improved PMS symptoms [24].

Mindfulness-based cognitive therapy (MBCT) was developed by Segal et al. in 2002. The aim of MBCT is for an individual to gain freedom from automatic reactions to thoughts, feelings, and events [25]. It emphasizes accepting thoughts and feelings without judgment. The skills taught in MBCT aim to help participants identify and accept negative thought patterns and respond in intentional ways [26]. In MBCT, a person accepts and welcomes tensions, stress, and pain, as well as disturbing emotions such as fear, anger, and feelings of unworthiness [27]. MBCT includes cognitive therapy and mindfulness skills. It consists of teaching participants various stress management techniques, including relaxation, yoga, and self-care techniques, in a systematic way. MBCT also uses meditation practice to increase attention and awareness. In MBCT, participants are encouraged to use “mind management skills” like breathing and bodily sensations to diminish goal-oriented thinking (conceptualizing of mind) in which emotions are experienced without awareness [27].

Although previous studies have shown that MBCT reduces depression and anxiety in women [28], few studies have examined the effectiveness of MBCT in women with PMS. Bluth et al. conducted a pilot study for the effectiveness of MBCT on PMDD. They reported that MBCT training reduced symptom severity for 7 of the 11 premenstrual symptoms [29]. To date, no randomized, controlled, prospective trial assessing the effectiveness of MBCT in patients with PMS has been published. Previous research has shown that women with severe PMS exhibit dysregulation in cardiovascular stress reactivity [30] and greater sensitivity to pain stimuli than women without severe PMS [31]. Consequently, we hypothesized that MBCT may improve sensitivity to pain, mood irritability, and stress reactivity in women with PMS. Previous studies have emphasized that MBCT has the ability to modulate stress and emotion regulation [32] by focusing on the realization that most thoughts and emotions fluctuate or are transient [33]. It seems that a release from fluctuating thoughts and emotions improves the regulation of emotional affect. We believe that when fluctuation of emotion is resolved, pain and stress sensitivity decreases, and symptoms of PMS improve. Previous researches have mentioned the effectiveness of MBCT in pain condition [34, 35]. In this study, we examined the effects of MBCT in patients with PMS who had depressive symptoms. The effects of MBCT on PMS, depression, and anxiety symptoms were investigated.

## 2. Method

### 2.1. Participants and Process

This was a randomized controlled clinical trial conducted at Mazandaran University from January to June 2015. The study was a thesis for a Master's degree in Clinical Psychology. All aspects of this protocol were approved by the Humanistic Science Committee of Islamic Azad University of Ayatollah Amoli.

After receiving permission from the dean of faculty to distribute the questionnaires, multistage cluster sampling was used to recruit students at Mazandaran University (located in Babolsar in the north of Iran) based on their field and academic year. Four hundred students were selected from a total enrolment of 3,000 students in four faculty clusters (Engineering, Humanities, Art, and Basic Sciences). Four sampling units were selected based on academic year of the students (first to fourth year). One hundred students were randomly selected for each sampling unit. Inclusion criteria were normal menstruation for at least two years, willingness to participate in the study, a diagnosis of PMS and mild or moderate depression (Beck score 16–47), and not currently taking any psychotherapy, not using any support group or relaxation technique, and not taking any antidepressant drugs. Women with no depressive symptoms (BDI score less than 16), severe depressive symptoms (BDI greater than 47), or PMDD (exhibiting more than five of the following symptoms: depressed mood, hopelessness, humiliation, anxiety, tension, irritability, excitement, mood swings, withdrawal from people, marked anger, increased conflict, and restlessness) were excluded from the study. A female researcher conducted a structured invitation with potential participants. The researcher explained the project to female students who fulfilled the inclusion criteria. A total of 400 female students were invited for the study. One hundred women refused to enter the project because they did not want to complete the questionnaires. The remaining 300 participants accepted the invitation to enter the screening plan.

After completing the demographic questionnaire, participants completed the Premenstrual Assessment Scale (PAS) for diagnosis and severity of PMS for at least two menstrual cycles. The students were asked to complete the PAS one week before and after menstruation [17]. A diagnosis of PMS was confirmed with at least two symptoms (one physical and one psychological), according to the American College of Obstetricians and Gynecologists criteria [17]. The participants also completed the Beck Depression Inventory (BDI). Over half (165 of 300) of the women were diagnosed with PMS and mild to moderate depressive syndrome. Students with severe depressive symptoms or PMDD were referred to an appropriate psychiatry service and excluded from the study. Of the eligible students, only 60 agreed to participate in the study. All participants signed a statement of informed consent and were assigned randomly into one of two groups (experimental, *n* = 30, and control, *n* = 30). Previous studies show that psychotherapy will reduce the PMS symptoms by 30%. A sample size of 30 in each group is required (*α* = 0.05, power 80%). Block randomization was done based on paper list. Random numbers were supplied from 1 to 60 by the trial statistician and prepared by an investigator with no clinical involvement in the trial. Odd numbers were assigned to the experimental group and even numbers to the control group. Serial evaluations of the patients' symptoms were performed by a researcher who was blinded to the treatment status of the patients, using three questionnaires at the beginning of the study (baseline) and after treatment (8 weeks after baseline). In the beginning of the study, all participants were asked to complete the PAS, BDI, and Beck Anxiety Inventory (BAI). Demographic characteristics such as age, educational level, marital status, and grade in university were ascertained at baseline.

The experimental group received MBCT in eight group sessions (120 min each) over 8 weeks. Each group consisted of 8–12 participants. A female therapist trained in MBCT by a supervisor (M. Faramarzi) before the trial conducted the sessions. The MBCT program consisted of integrating elements of mindfulness-based stress reduction and CBT with guided depression/anxiety meditations. The program drew on traditional mindfulness meditation techniques, as well as guided meditation (daily activity related to depression/anxiety), to address specific issues pertaining to depression/anxiety. Patients in the control group did not receive any intervention

The following list is a model treatment outline of mindfulness-based cognitive therapy sessions for women with premenstrual syndrome based on the work by Kabat-Zinn [36].

Session 1: building a therapeutic alliance and obtaining information from the client, identifying automatic thoughts, introducing the body scan, raisin exercise, and introducing mindfulness meditation with in-session practice. Assignment: reading about the body scan meditation technique, 30-minute daily formal practice (body scan meditation), informal practice, and awareness to some routine activity such as washing dishes or eating a meal (continued throughout trial period).
Session 2: helping the client recognize that thoughts are not facts, teaching use of the thought record, sitting meditation using breath as the primary object of awareness, and alternating this with the body scan (sitting one day, body scan the next, etc.). Assignment: reading about and doing formal and informal sitting meditation.
Session 3: dealing with automatic thoughts in life and in meditation and walking meditation. Assignment: mindful yoga.
Session 4: stopping one-minute breathing space. Assignment: mindful yoga and sitting meditation (continued throughout trial period).
Session 5: dealing with difficult emotions, wisdom meditation, and walking meditation. Assignment: mindful yoga.
Session 6: communication. Assignment: listening to others carefully and mindful yoga.
Session 7: self-compassion. Assignment: loving yourself and mindful Yoga.
Session 8: helping the client develop a practice of her own, reviewing progress, insights, and techniques, and individual evaluation of the sessions.

MBCT is conceptualized as a way of increasing the awareness of automatic patterns and then to disengage undesirable reactivity [36]. For individual practice, participants read printed copies of material about an important part of the program, did daily formal practice for 30 minutes, did informal practice, and listened to a 20–60-minute prerecorded CD two times daily over a period of 8 weeks [37]. At the beginning of each session, the therapist asked the patients to do mindfulness skills during class and described one person's individual experience with mindfulness out of class. The therapist then helped the patients to archive the corrected mindfulness skills.

### 2.2. Measures

#### 2.2.1. Premenstrual Assessment Scale (PAS)

The PAS is a self-reported questionnaire designed in an Iranian project. It contains 32 items and two subscales that cover somatic and psychological symptoms. Each item is scored from 0 to 3 (0: no symptom, 1 mild, 2 moderate, and 3 severe). The PAS has a validity of 0.92 and a reliability of 0.84 [38].

#### 2.2.2. Beck Depression Inventory (BDI)

The 21-item BDI describes specific behavioral manifestations of depression. Each item can be scored from 0 (no depressive symptoms) to 3 (severe level of symptoms). The total score is determined by summing the individual scores and ranges from 0 to 63. Depression scores are classified as follow: ≤15, normal to minimal depressive symptoms; 16–31, mild depression; 32–47, moderate depression; and >47, severe depression [39]. We used the validated Persian 21-BDI [40].

#### 2.2.3. Beck Anxiety Inventory (BAI)

The BAI is comprised of 21 symptoms that measure anxiety levels. Each item can be scored from 0 (not at all) to 3 (severely). The total score is determined by summing the individual scores and ranges from 0 to 63. The BAI has a high internal consistency and test-retest reliability [41]. The Persian version of the BAI has previously shown good reliability and validity (*r* = 0.83, *p* < 0.001) and excellent internal consistency (Cronbach's alpha = 0.92) [42].

### 2.3. Statistical Calculations

The results were analyzed using repeated-measure ANOVAs, with 2 times (pretest treatment, posttest) as a within-subjects factor (MBCT and control) as a between-subjects factor. This model was used for all the dependent variables (depression, anxiety, and Premenstrual Syndromes) to assess whether the interaction effect time × group was significant.

## 3. Results

The demographic data are presented as frequency and percentage in Table 1. The majority of the participants were undergraduate students (94%), with 6% at the Master's level. Of the undergraduates, 35% were seniors, 31% juniors, and 19% sophomores. There were no significant differences between the control and intervention groups at baseline in sociodemographic or clinical characteristics.

Table 2 shows the pre- and postintervention means and standard deviations for all measures. Repeated-measure ANOVAs on depression revealed a significant interaction effect for group × time. There was difference between the MBCT and control group in terms of the improvement depression symptoms at posttest (*F*[1,58] = 7.80; *p* = 0.007). ANOVAs on each group over time of the trial revealed that the MBCT group improved the mean scores of depression symptoms significantly from pretreatment to posttreatment (*p* < 0.001). Repeated-measure ANOVAs on anxiety revealed a significant interaction effect for group × time. There was difference between the MBCT and control group in terms of the improvement anxiety symptoms at posttest (*F*[1,58] = 7.30; *p* = 0.007). ANOVAs on each group over time of the trial revealed that the MBCT group improved the mean scores of anxiety symptoms significantly from pretreatment to posttreatment (*p* < 0.001). Control group did not improve depression and anxiety symptoms from pretreatment to posttreatment.

Repeated-measure ANOVAs on symptoms of PMS revealed a significant interaction effect for group × time. There was difference between the MBCT and control group in terms of the improvement symptoms of PMS at posttest (*F*[1,58] = 17.02; *p* ≤ 0.001). ANOVAs on each group over time of the trial revealed that the MBCT group improved the mean scores of symptoms of PMS significantly from pretreatment to posttreatment (*p* < 0.001). Control group did not improve symptoms of PMS from pretreatment to posttreatment.

## 4. Discussion

Our study is the first to show that MBCT improves depression, anxiety, and symptoms of PMS. Few published studies have reported the effects of psychotherapy on depression, anxiety, symptoms of PMS, and no previous studies have used a randomized controlled trial.

The improvement of PMS symptoms after MBCT is in line with previous research. Morse et al. reported that CBT with 10 weekly 1-hour group sessions reduced the symptoms of PMS by 5 weeks after test [43]. Kirkby compared 48 women with PMS in a CBT (six weekly 10-hour group sessions) or control (waitlist) group. He observed that CBT reduced anxiety, depression, and premenstrual symptoms [44]. Blake et al. observed that CBT reduced psychological but not somatic symptoms in women with PMS [45].

Some reports disagree with our findings. In the study by Morse et al., 42 Australian women with menstrual disorders in three groups were assigned to a progesterone, CBT, or relaxation group. They concluded that symptoms of anxiety, depression, and PMS improved for the progesterone and relaxation groups, but not the CBT group [46]. Christensen and Oei compared the effects of two kinds of intervention on improving Premenstrual Dysphasia and concluded that 13 weekly 20-hour group sessions of CBT did not reduce symptoms of depression, anxiety, or PMS [47]. Hunter et al. (2002) studied 108 women with PMDD and reported that interventions such as CBT (8 1-hour sessions over three months) did not improve the symptoms of PMDD. At a one-year follow-up, the CBT group had significantly fewer participants with PMDD symptoms than did other psychotherapy treatment groups [22]. In a systematic review, Lustyk et al. investigated seven randomized controlled studies to determine the effectiveness of CBT in women with PMS or PMDD. They reported a dearth of evidence providing statistically significant CBT intervention effects [23].

We saw a large effect of MBCT on PMS symptoms and anxiety, and depression symptoms in women with PMS. This has not been reported in previous studies on other psychotherapies (such as CBT), which may be attributable to the natures of both the therapy and the PMS condition. Although this is the first randomized controlled study to apply MBCT to women with PMS, we believe that the substantial effect it was found to have on the PMS symptoms, anxiety, and depression may be related to the nature of the method. Anxiety and depression play an important role in increased symptoms in women with PMS [13]. Previous studies provide strong support for the efficacy of MBCT for the treatment of depressive/anxiety disorders [28, 48]. As with many applications of mindfulness and related meditation techniques, framing MBCT within a relaxation or stress coping context can play a large role in improving outcomes. As psychosocial factors predict stress [49] and stress is a common trigger of PMS symptoms [12], this is particularly true in relation to PMS. Another key aspect of MBCT involves helping patients recognize how anxious or depressive thoughts may exacerbate their PMS symptoms. MBCT provides individuals with a heightened ability to simply observe thoughts, feelings, and experiences in order to disengage automatic and often dysfunctional reactivity and then to allow them to work with more balanced relationships with themselves. This linking process may be an important key of the therapeutic mechanism. A release from anxiety/depression symptoms may improve the regulation of emotional affect and have central healing effects on women with PMS. Thus, it seems that coordination between the method of MBCT and the nature of PMS may contribute to the successful results of psychotherapy.

Due to some limitations, generalizations of our results should be made with caution. First, the sample size was small, and we recommend further research with a larger sample. Second, the MBCT group received more treatment, and the positive results obtained may be due to the additional treatment (i.e., more contact) rather than anything specific about MBCT. Further research is needed with a control group of women with PMS who are receiving placebo group therapy. Moreover, further research is necessary to compare the effect of MBCT with CBT in women with PMS. Third, our results were self-reported. Forth, the subjects were not blind to their treatment, and this may have been amplified by a recruitment bias. Since the subjects knew in advance that the study would explore the effect of mindfulness on their symptoms, it is possible that those who accepted anticipated a beneficial effect and thus already believed in the benefits of mindfulness. This may have affected the way they rated their symptoms at the end of the study. Further research is needed to assess the change with a placebo group and also with a physician to distinguish between physical and subjective symptoms. Fifth, MBCT has component of CBT as well as relaxation. Therefore, it cannot be concluded with confidence that it is a component specific to MBCT that has led to the changes after intervention, in view of a lack of measure of changes in mindfulness skills in this study. Also, we did not use the measurement of homework compliance although there were regular homework assignments for the participants.

In conclusion, MBCT improved PMS symptoms, anxiety, and depression, in women with Premenstrual Syndrome. These results may be important for obstetricians, midwives, nurses, and other health professionals. This study supports implications for psychotherapy in women with PMS who suffer from symptoms of anxiety or depression. Furthermore, an economic evaluation of the addition of psychological intervention to medical therapy in women with mild to moderate PMS would be useful.

## Figures and Tables

**Table 1:** Demographic characteristic of the students.

Variables	Experimental group *N* (%)	Control group *N* (%)
Age (years)		
≤20	8 (26.6)	6 (20.0)
>20	22 (73.4)	24 (80.0)
Marital status		
Single	26 (86.6)	25 (83.3)
Married	4 (13.4)	5 (16.7)
Grade level		
Freshman	4 (13.3)	4 (13.3)
Sophomore	5 (16.7)	6 (20.0)
Junior	13 (43.3)	6 (20.0)
Senior	8 (26.7)	14 (46.7)

**Table 2:** Mean and standard deviation of variables in two groups in beginning of and after intervention.

Variables	Experimental group		Control group		*p* value
	Pretest Mean (SD)	Posttest Mean (SD)	Pretest Mean (SD)	Posttest Mean (SD)	
Depression (total BDI)	24.70 (7.06)	15.73 (6.99)	24.96 (7.17)	25.36 (7.14)	0.007
Anxiety (total BAI)	26.53 (7.22)	16.96 (7.78)	27.56 (7.32)	26.60 (9.38)	0.007
PMS (total PAS)	58.66 (7.62)	42.86 (8.02)	58.28 (7.50)	58.93 (8.47)	<0.001
